# The complete mitochondrial genome of Pink warty sea cucumber (*Cercodemas anceps* Selenka, 1867)

**DOI:** 10.1080/23802359.2021.1891979

**Published:** 2021-03-18

**Authors:** Huo Li, Jinshang Liu, Shengpeng Wang, Wen Huang

**Affiliations:** a Guangdong Provincial Engineering and Technology Research Center, Doctoral Workstation of Guangdong Province, Guangdong Jinyang Biotechnology co. LTD, Maoming, China; b Laboratory of Aquatic Sciences, Key Laboratory of Animal Nutrition and Feed Science in South China of Ministry of Agriculture and Rural Affairs, Guangdong Key Laboratory of Animal Breeding and Nutrition, Institute of Animal Science, Guangdong Academy of Agricultural Sciences, Guangzhou, China

**Keywords:** Mitochondrial genome, Holothuroidea, *Cercodemas anceps*, phylogenetic analysis

## Abstract

In this study, we sequenced the circular mitochondrial genome (mitogenome) of *Cercodemas anceps*. This genome was determined to measure 16,539 bp in length and contain 13 protein-coding genes (PCGs), 22 tRNA genes, and 2 rRNA genes. The longest gene was observed to be *nad5*, which measures 1,641 bp in length and is located at position 6,540 ∼ 8,180 of the *C. anceps* mitogenome. One PCG, *nad6*, and five tRNA genes (*tRNA^Ser(UCN)^*, *tRNA^Gln^*, *tRNA^Ala^*, *tRNA^Val^,* and *tRNA^Asp^*) were located on the light chain, and the other genes were located on the heavy chain. A phylogenetic tree was constructed with the mitogenome sequences of 26 types of echinoderm species, and the results show that *C. anceps* is most closely related to *C. quadrangularis*.

Sea cucumbers play important roles in maintaining healthy coral reef ecosystems (Birkeland 1989; Schneider et al. 2011; Huang et al. 2018). *Cercodemas anceps* (Cucumariidae) primarily grows in the coastal shallow waters of the South China Sea (Liao 1997). Live *C. anceps* exhibits bright yellow and red colors (Lane et al. 2000), which causes this species to be a prime target for recreational fisheries in China. Li (a local fisherman in Sanya city, Hainan Province, China) stated that the average price (in 2019) of each live Pink warty sea cucumber was approximately US$8.

The highly conservative pattern of the mitochondrial genome (mitogenome) and its rapid rate of evolution makes the mitogenome an ideal tool for studying evolution and molecular ecology (Yoon et al. 2006; Verbruggen et al. 2010; Janouškovec et al. 2013), and this genome is generally considered as to be a useful molecular marker for phylogenetic analyses and species identification (Byrne et al. 2010; Zhang et al. 2015).

In the present study, *C. anceps* was obtained from Sanya Cape (N18°2021′, E109°4708′), Sanya city, Hainan Province, China. Total DNA was extracted with the TIANamp Marine Animal DNA Kit (TIANGEN, Beijing, China) and stored with the sample ID GDAAS-IAS-AQUA-CA-202006125 at Guangdong Academy of Agricultural Sciences (Guangzhou, China). Libraries with average lengths of 350 bp were constructed using the NexteraXT DNA Library Preparation Kit (Illumina, Shanghai, China) and sequenced on an Illumina HiSeq 4000 sequencing platform (paired-end 150-bp reads were generated) at Shenzhen Huitong Biotechnology Co. Ltd, China. Raw sequence reads were edited using the NGS QC Tool Kit v2.3.3 and assembled into contigs using the *de novo* assembler SPAdes 3.11.0 (Dmitry et al. 2016), and the complete mitogenome was deposited on the NCBI website (https://www.ncbi.nlm.nih.gov/genbank/) with the accession number MW044622.1. In total, 15 reported sea cucumber species were selected, and the phylogenetic relationships were reconstructed with the protein-coding genes by means of maximum-likelihood (ML) (GTR + G + I model) analysis using MEGA-X software (Kumar et al. 2018) with 1000 replicates. Ten types of other echinoderm mitogenome sequences were utilized as outgroups.

The mitogenome of *C. anceps* was determined to be 16,539 bp long (41.86% A, 27.74% T, 18.74% C, and 11.66% G) and to include a set of 22 tRNA genes, 13 protein-coding genes (PCGs), and 2 rRNA genes. *nad5* was observed to be the longest gene, measuring 1641 bp in length, and it was located at position 6540–8180 of the *C. anceps* mitogenome. The lengths of the tRNA genes ranged from 65 bp to 74 bp, and PCGs ranged from 159 bp to 1641 bp. One PCG (*nad6*) and five tRNA genes (*tRNA^Ser(UCN)^*, *tRNA^Gln^*, *tRNA^Ala^*, *tRNA^Val^,* and *tRNA^Asp^*) were located on the light chain, and the other genes were located on the heavy chain.

A phylogenetic analysis showed that *C. anceps* is most closely related to *C. quadrangularis*, and other Cucumariidae species (*Cucumaria miniata*, *Pseudocolochirus violaceus*, and *Colochirus quadrangularis*) (Figure 1). The results of this investigation of the *C. anceps* mitogenome contributes to the growing data on holothuroid mitogenomes and provides useful information for phylogenetic and evolutionary studies in the selected sea cucumber species.

**Figure 1.**
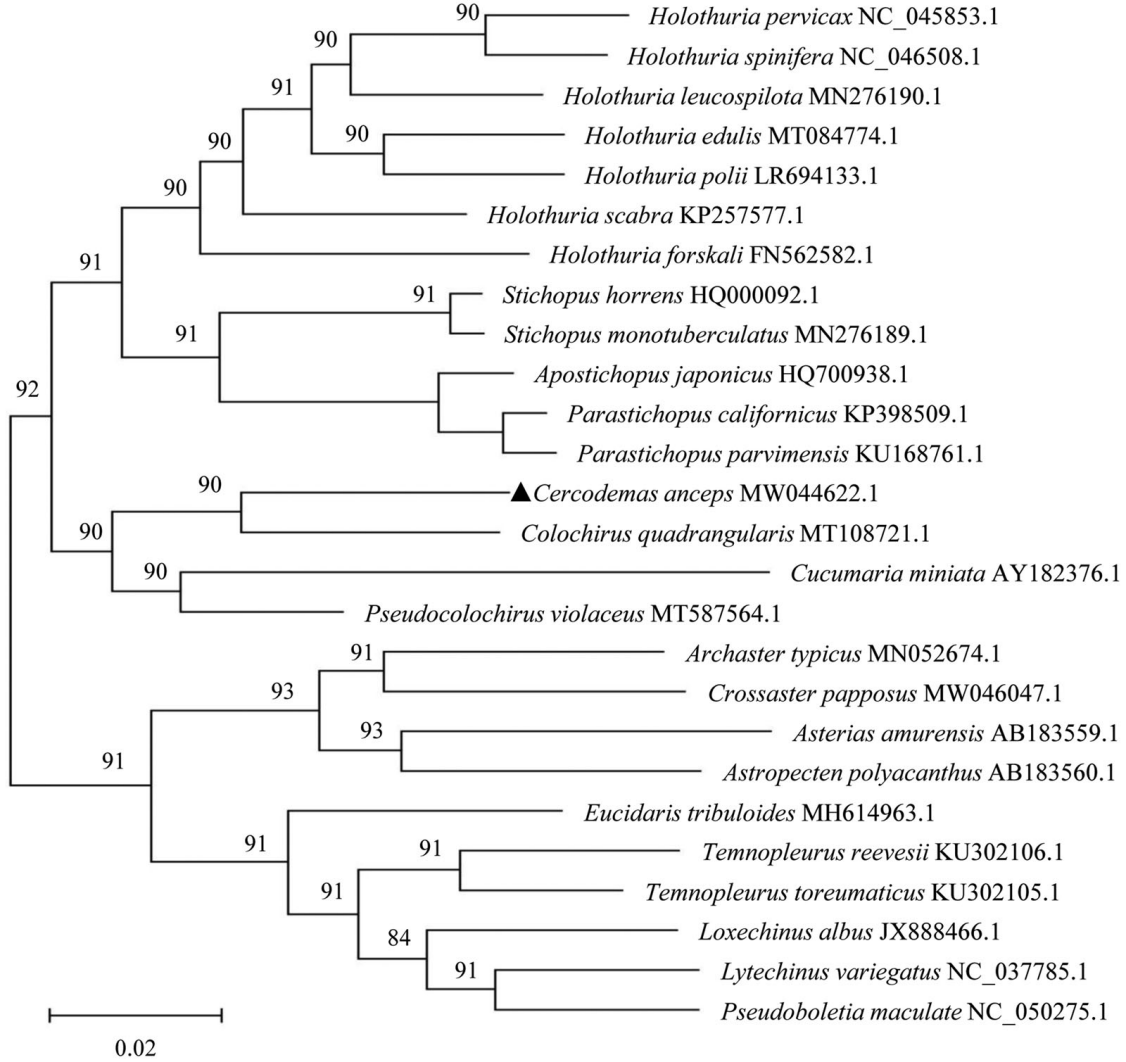
Maximum likelihood (ML) phylogenetic tree based on complete mitochondrial genomes of 26 species. Values along branches correspond to ML bootstrap percentages. The phylogenetic position of *Cercodemas anceps* was marked with a dark-triangle.

## Data Availability

The genome sequence data that support the findings of this study are openly available in GenBank of NCBI at (https://www.ncbi.nlm.nih.gov/) under the accession no. MW044622.1. The associated BioProject, SRA, and Bio-Sample numbers are PRJNA690177, SRR13385161, and SAMN17198833, respectively.

## References

[CIT0001] Birkeland C. 1989. The influence of echinoderms on coral-reef communities. Echinoderm Stud. 3:1–79.

[CIT0002] Byrne M, Rowe F, Uthicke S. 2010. Molecular taxonomy, phylogeny and evolution in the family Stichopodidae (Aspidochirotida: Holothuroidea) based on COI and 16S mitochondrial DNA. Mol Phylogenet Evol. 56(3):1068–1081. 20399872 10.1016/j.ympev.2010.04.013

[CIT0004] Dmitry A, Anton K, Jeffrey SM, Pavel AP. 2016. HYBRIDSPADES: an algorithm for hybrid assembly of short and long reads. Bioinformatics. 32:7. 10.1093/bioinformatics/btv688 PMC4907386 26589280

[CIT0005] Huang W, Huo D, Yu Z, Ren C, Jiang X, Luo P, Chen T, Hu C. 2018. Spawning, larval development and juvenile growth of the tropical sea cucumber *Holothuria leucospilota*. Aquaculture. 48:22–29.

[CIT0006] Janouškovec J, Liu SL, Martone PT, Carré W, Leblanc C, Collén J, Keeling P. 2013. Evolution of red algal plastid genomes: ancient architectures, introns, horizontal gene transfer, and taxonomic utility of plastid markers. PLoS One. 8(3):e59001. 23536846 10.1371/journal.pone.0059001 PMC3607583

[CIT0007] Kumar S, Stecher G, Li M, Knyaz C, Tamura K. 2018. MEGA X: molecular evolutionary genetics analysis across computing platforms. Mol Biol Evol. 35(6):1547–1549. 29722887 10.1093/molbev/msy096 PMC5967553

[CIT0008] Lane DJW, Marsh LM, VandenSpiegel D, Rowe FWE. 2000. Echinoderm fauna of the South China Sea: an inventory and analysis of distribution patterns. Raffles Bull Zool. 48(8 SUPPL.):459–493.

[CIT0009] Liao Y. 1997. Fauna Sincia: Phylum Echinodermata Class Holothuroidea. Beijing: Science Press.

[CIT0010] Schneider KJ, Silverman E, Woolsey H, Eriksson M, Byrne K. Caldeira 2011. Potential influence of sea cucumbers on coral reef CaCO3 budget: a case study at One Tree Reef. J Geophysical Research: Biogeo Sci. 116:1–6.

[CIT0011] Verbruggen H, Maggs CA, Saunders GW, Le Gall L, Yoon HS, Clerck OD. 2010. Data mining approach identifies research priorities and data requirements for resolving the red algal tree of life. BMC Evol Biol. 10:16. 20089168 10.1186/1471-2148-10-16 PMC2826327

[CIT0012] Yoon HS, Müller KM, Sheath RG, Ott FD, Bhattacharya D. 2006. Defining the major lineages of red algae (Rhodophyta). J Phycol. 42(2):482–492.

[CIT0013] Zhang HX, Zhang YH, Qin G, Lin Q. 2015. The complete mitochondrial genome sequence of the network pipefish (*Corythoichthys flavofasciatus*) and the analyses of phylogenetic relationships within the Syngnathidae species. Mar Genomics. 19:59–64. 25500378 10.1016/j.margen.2014.11.005

